# Stochastic analysis of the *GAL *genetic switch in *Saccharomyces cerevisiae*: Modeling and experiments reveal hierarchy in glucose repression

**DOI:** 10.1186/1752-0509-2-97

**Published:** 2008-11-17

**Authors:** Vinay Prasad, KV Venkatesh

**Affiliations:** 1 Department of Chemical Engineering, Center for Catalytic Science and Technology, University of Delaware, Newark, DE 19716-3110, USA; 2 Department of Chemical Engineering, Indian Institute of Technology, Bombay, Powai, Mumbai, 400076, India

## Abstract

**Background:**

Transcriptional regulation involves protein-DNA and protein-protein interactions. Protein-DNA interactions involve reactants that are present in low concentrations, leading to stochastic behavior. In addition, multiple regulatory mechanisms are typically involved in transcriptional regulation. In the *GAL *regulatory system of *Saccharomyces cerevisiae*, the inhibition of glucose is accomplished through two regulatory mechanisms: one through the transcriptional repressor Mig1p, and the other through regulating the amount of transcriptional activator Gal4p. However, the impact of stochasticity in gene expression and hierarchy in regulatory mechanisms on the phenotypic level is not clearly understood.

**Results:**

We address the question of quantifying the effect of stochasticity inherent in these regulatory mechanisms on the performance of various genes under the regulation of Mig1p and Gal4p using a dynamic stochastic model. The stochastic analysis reveals the importance of both the mechanisms of regulation for tight expression of genes in the *GAL *network. The mechanism involving Gal4p is the dominant mechanism, yielding low variability in the expression of *GAL *genes. The mechanism involving Mig1p is necessary to maintain the switch-like response of certain *GAL *genes. The number of binding sites for Mig1p and Gal4p further influences the expression of the genes, with extra binding sites lowering the variability of expression. Our experiments involving growth on various substrates show that the trends predicted in mean expression and its variability are transmitted to the phenotypic level.

**Conclusion:**

The mechanisms involved in the transcriptional regulation and their variability set up a hierarchy in the phenotypic response to growth on various substrates. Structural motifs, such as the number of binding sites and the mechanism of regulation, determine the level of stochasticity and eventually, the phenotypic response.

## Background

It is well known that gene expression is a highly stochastic, or noisy, process [1]. The cause of this stochasticity lies in the fact that many components are present in low concentrations within a cell. When low numbers of molecules are present, continuum rate expressions based on mass action kinetics are no longer valid. For simple systems, consisting of the expression of 1–2 genes, the stochasticity has been characterized as 'intrinsic noise' [1,2]. Fluctuations in the states of other cellular components may also affect the gene expression indirectly, and this effect is classified as 'extrinsic noise'. However, in real systems composed of multiple genes with multiple interactions, it is of primary importance to study and quantify the effect of the stochasticity due to intrinsic noise, and separate its effect from that of extrinsic noise [3,4]. For well-studied systems where the interactions are known, intrinsic noise can be computed using simulation methods such as the Stochastic Simulation Algorithm (SSA) of Gillespie [5], and other exact and approximate stochastic simulation methods [6-13]. One such system is the *GAL *network of *Saccharomyces cerevisiae*. In this work, we characterize the intrinsic noise of the GAL network in response to variations in glucose concentration.

**Figure 1:**
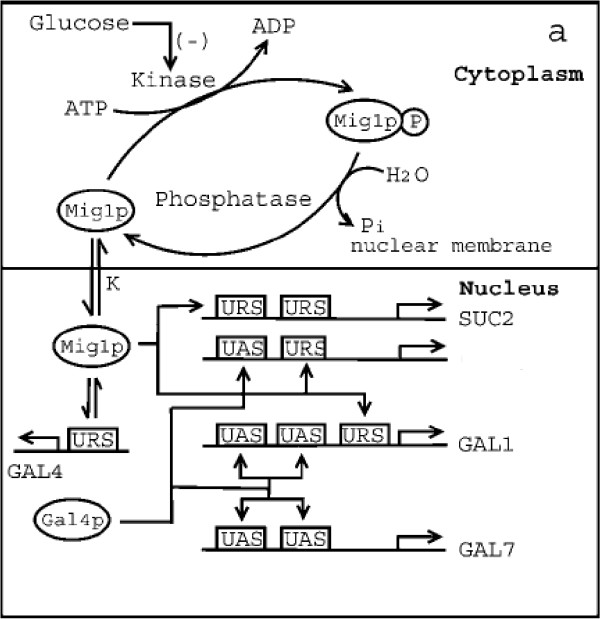
**A schematic of the glucose inhibition of the *GAL *regulatory system in a mutant strain of *Saccharomyces cerevisiae *lacking *GAL80*.** It should be noted that *GAL2 *and *GAL7 *share a similar mechanism of regulation with two binding sites for Gal4p. *MEL1 *and *GAL3 *also share a similar mechanism with one binding site for Gal4p and Mig1p.

The *GAL *system codes for genes that are responsible for protein expression involved in the Leloir pathway (see Figure 1 for the schematic). The *GAL *network of *S. cerevisiae *is activated by galactose and inhibited by glucose. In a wildtype strain, Gal4p is a transcriptional activator whose synthesis is regulated by glucose concentration. The synthesis is repressed at high glucose concentrations. The activity of Gal4p as a transcriptional activator is controlled by a repressor, Gal80p, which is also a member of the *GAL *system. Gal3p, a galactose sensor, binds to Gal80p to release its effect on Gal4p. Thus, in the presence of galactose, Gal3p and Gal80p are bound to each other, and this allows the free Gal4p to bind to the upstream activating sequence (UAS) to express *GAL *genes. The binding of Gal3p and Gal80p is initiated by intracellular galactose. The amount of intracellular galactose is controlled by the amount of permease Gal2p (synthesized by *GAL2 *gene), which transports it from the extracellular medium. However, in the presence of glucose, a kinase (Mig1p) binds to the upstream repressing sequence (URS) of certain *GAL *genes and *GAL4 *to repress their synthesis. Mig1p is a constitutively expressed [14] global repressor protein, whose activity is regulated through a phosphorylation-dephosphorylation cycle [15-18]. In the presence of glucose, it is believed that Snf1 kinase (a homologue of ADP-AMP kinase in humans) is inactivated through a mechanism that is not clearly understood [16,17]. Under these conditions, Mig1p is predominantly in the dephosphorylated state and translocates into the nucleus [17,19] to repress genes by binding to the URS of various genes. In *S. cerevisiae*, three different mechanisms can be observed for glucose repression through Mig1p. Expression is repressed directly by binding of Mig1p to the URS of genes such as *SUC2 *and *GAL4 *[14,20,21]. In the *GAL/MEL *regulon, Mig1p represses the structural (such as *MEL1*, *GAL1*, *GAL7*) and regulatory (such as *GAL3*, *GAL80*) genes indirectly through Gal4p. In this case, Mig1p represses only the expression of the activator, and thus indirectly represses the structural genes (like in *GAL2 *and *GAL7*) [22]. In addition, a set of structural genes (*GAL1, MEL1*) as well as a regulatory gene (*GAL3*) have a URS for Mig1p binding as well as an upstream activation sequence (UAS) for the transcriptional activator [15,20,21]. Intuitively, by repressing the genes through a common activator such as Gal4p, the cell achieves the repression in a coordinated fashion, instead of repressing each gene through an independent URS. However, the above reason alone does not explain why only a few genes are repressed through an activator. An analysis based on steady state modeling of the Mig1p dependent repression revealed that a transcriptional hierarchy could be established solely through the various mechanisms existing for glucose repression, without sacrificing amplification and sensitivity [22].

Stochastic analysis of the *GAL *system has also been reported; however, studies have focused on the response of the system to galactose (inducer), and on the role of the Gal3p and Gal80p regulatory mechanisms [23-26]. Other studies include a study of the transcriptional regulation of the metabolism of galactolysis and glucolysis and their integration with the *GAL *genetic network [27]. In the current study, to analyze the role of various mechanisms of glucose repression on the stochastic behavior of the *GAL *network, we consider a mutant strain of *S. cerevisiae *lacking Gal80p. It should be noted that in the absence of Gal80p, the activity of the transcriptional activator Gal4p is solely controlled through the effect of glucose concentration. Further, the role of Gal3p is also negated due to the absence of Gal80p; thus, such a mutant strain will constitutively express *GAL *genes even in the absence of galactose, and will respond only to variations in glucose concentration. The stochastic analysis reveals mechanisms for which the effect of inherent stochasticity is high. Both the regulators, Gal4p and Mig1p, are essential for complete repression by glucose with low noise. We also present experiments to determine whether the noise in the gene expression can be correlated to the variability at the phenotypic level.

## Results

**Figure 2:**
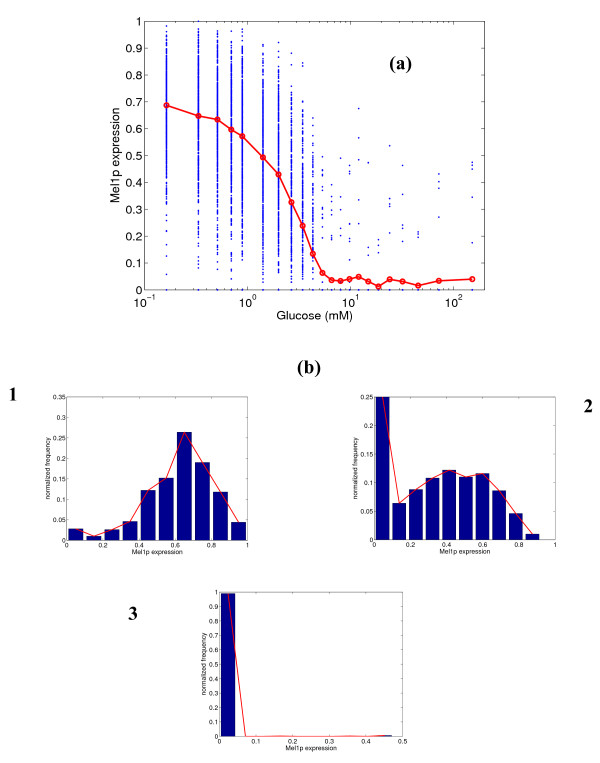
**(a) Simulated steady state protein expression for Mel1p at different glucose concentrations, and (b) distribution of expression at 0.33, 2.0 and 5.33 mM glucose.** In figure (a), the blue dots represent the range of expression values over 500 simulations, and the red line represents the mean expression.

The stochastic model was simulated to obtain steady state distributions of the *GAL *gene expression at various glucose concentrations. These distributions were computed as fractional gene expressions. The steady state distributions of fractional protein concentrations were computed from the fractional gene expression in the manner described in the section on simulation. Figure 2 shows the mean of the distributions for the expression of *MEL1 *gene. Shown in the figure are the expression of individual simulations (500 in all), and the mean expression from these simulations. We also present representative distributions of the expression across the simulations at three specific glucose concentrations (0.33, 2.0 and 5.33 mM). These distributions may be taken to represent the distributions in a cell population. We have chosen a low glucose concentration (high expression), an intermediate concentration in the sensitive region for expression, and a high glucose concentration indicating low expression for the distributions. The maximum mean fractional expression of *MEL1 *is 70%, which matches reasonably well with steady-state experimental data [22]. At lower glucose concentrations, the variability in the distribution is high (first histogram in panel 'b' of Figure 2). At high glucose concentrations, the variability is much lower (third histogram in panel 'b'). The variability decreases along with the level of expression as glucose concentration is increased. The sensitivity of the response, reported in terms of Hill's coefficient, is 1.7, which indicates a steep response.

**Figure 3:**
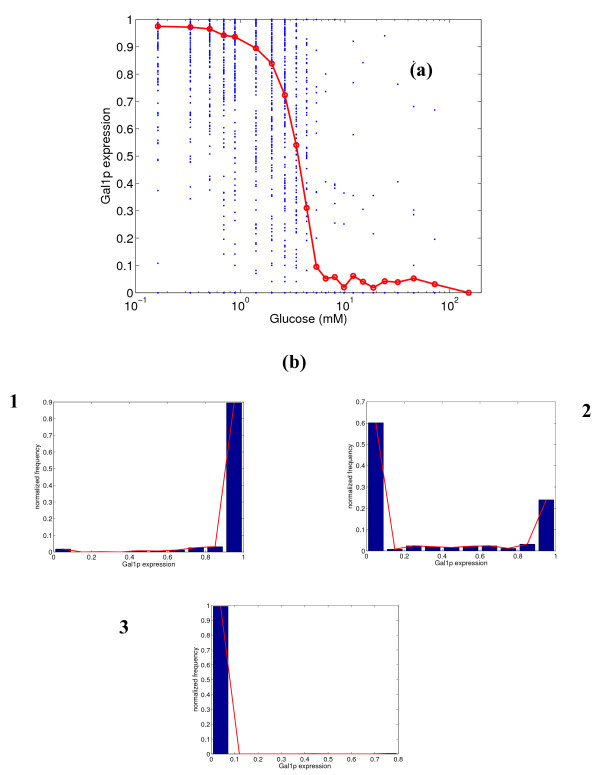
**(a) Simulated steady state protein expression for Gal1p at different glucose concentrations, and (b) distribution of expression at 0.33, 3.43 and 6.55 mM glucose.** In figure (a), the blue dots represent the range of expression values over 500 simulations, and the red line represents the mean expression.

**Figure 4:**
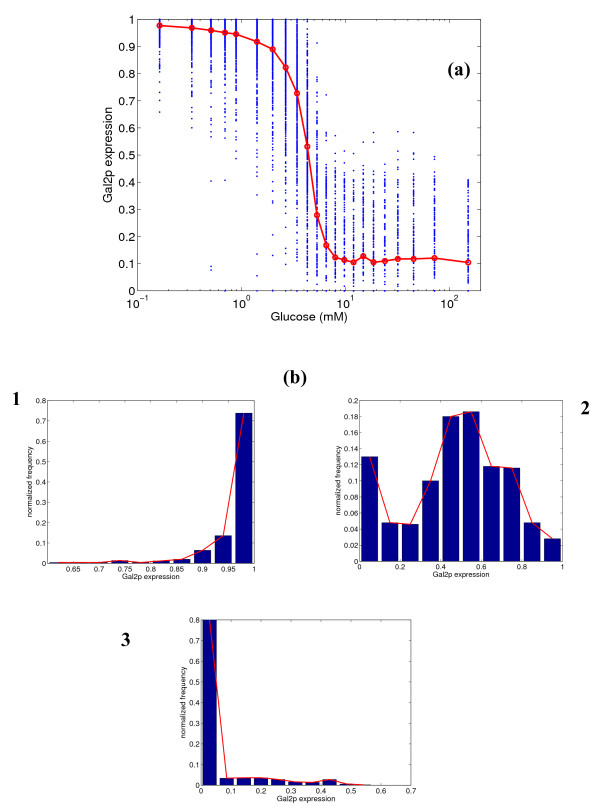
**(a) Simulated steady state protein expression for Gal2p at different glucose concentrations, and (b) distribution of expression at 0.33, 4.31 and 8.0 mM glucose.** In figure (a), the blue dots represent the range of expression values over 500 simulations, and the red line represents the mean expression.

Next, we conduct a similar analysis for the expression of *GAL1 *gene, and the results are shown in Figure 3. Gal1p is fully expressed at low glucose concentrations and almost completely repressed at high glucose concentrations, as has been demonstrated in experiments [22]. The variability is low at low and high glucose concentrations, and Gal1p demonstrates switch-like behavior at intermediate concentrations (see the panel of histograms in Figure 3b). The switch-like behavior is seen in the second histogram, where the frequency for intermediate expressions is very low, but higher frequency is observed at the extremes (close to 0 or 1 fractional expression). This is because of the steep nature of the response curve, indicative of a sensitive response (Hill's coefficient η_H _= 3.4). Figure 4 shows the expression of Gal2p, which is a permease that transports galactose from the extracellular medium. Gal2p has two binding sites for Gal4p and none for Mig1p, while Gal1p has one binding site for Mig1p along with two binding sites for Gal4p. Gal2p is fully expressed at low glucose concentrations, but has a leaky expression (mean = 10%) at high glucose concentrations. Also, the variability in the expression at intermediate glucose concentrations is relatively high, indicating a broad distribution. This is in direct contrast to the behavior of Gal1p. The Hill's coefficient is 2.5, much lower than that for Gal1p.

**Figure 5:**
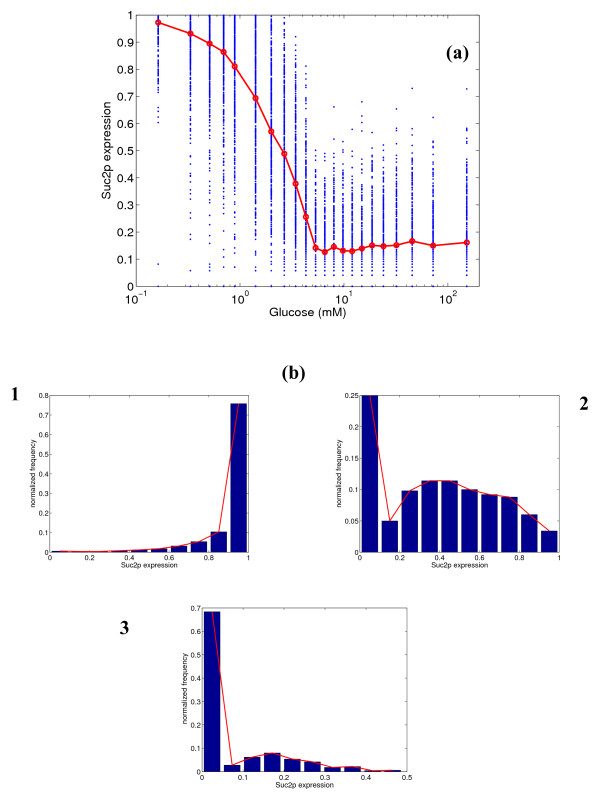
**(a) Simulated steady state protein expression for Suc2p at different glucose concentrations, and (b) distribution of expression at 0.33, 2.67 and 6.55 mM glucose.** In figure (a), the blue dots represent the range of expression values over 500 simulations, and the red line represents the mean expression.

Figure 5 shows the results for the expression of *SUC2 *gene at various glucose concentrations. Suc2p has two binding sites for Mig1p and none for Gal4p. Suc2p is also fully expressed at low glucose concentrations, but the response is leaky (mean = 15%) at high concentrations. The response is closer to a Michaelis-Menten type response, with η_H _= 1.5. The distribution shows similar characteristics to that of Gal2p.

**Figure 6:**
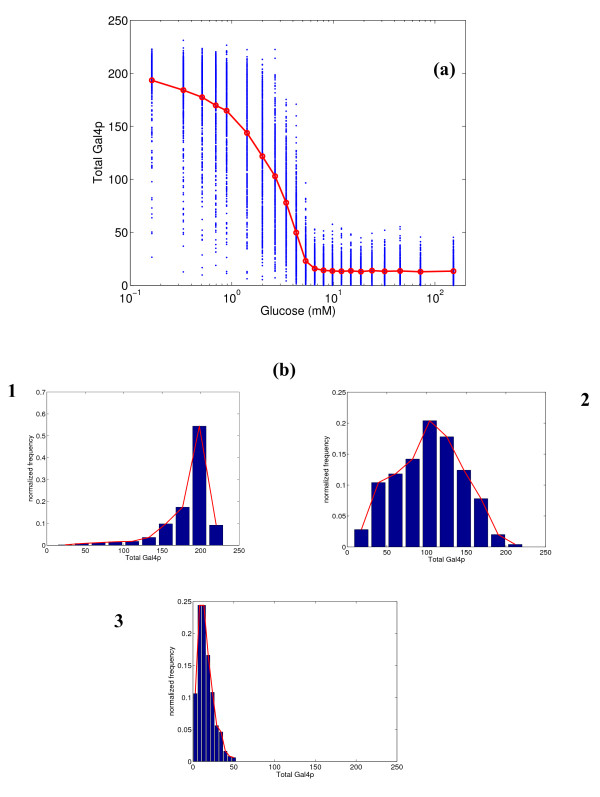
**(a) Simulated steady state protein expression for total Gal4p at different glucose concentrations, and (b) distribution of expression at 0.33, 2.67 and 6.55 mM glucose.** In figure (a), the blue dots represent the range of expression values over 500 simulations, and the red line represents the mean expression.

Figure 6 shows the variation of the total number of Gal4p molecules (in bound and unbound states) with respect to glucose concentration. Gal4p has one binding site for Mig1p. The mean number of molecules ranges from 200 at low glucose concentrations to 10 at high glucose concentrations. This corresponds to approximately 100 Gal4p dimers and 5 dimers at the two extremes. This is in agreement with experimental observations in the literature [28,20]. The Hill's coefficient is 1.7, which indicates higher sensitivity than SUC2, but lower than the other *GAL *genes. Unlike other *GAL *genes, Gal4p demonstrates relatively broad distributions at all glucose concentrations, with the variability being highest at the intermediate concentrations.

**Figure 7:**
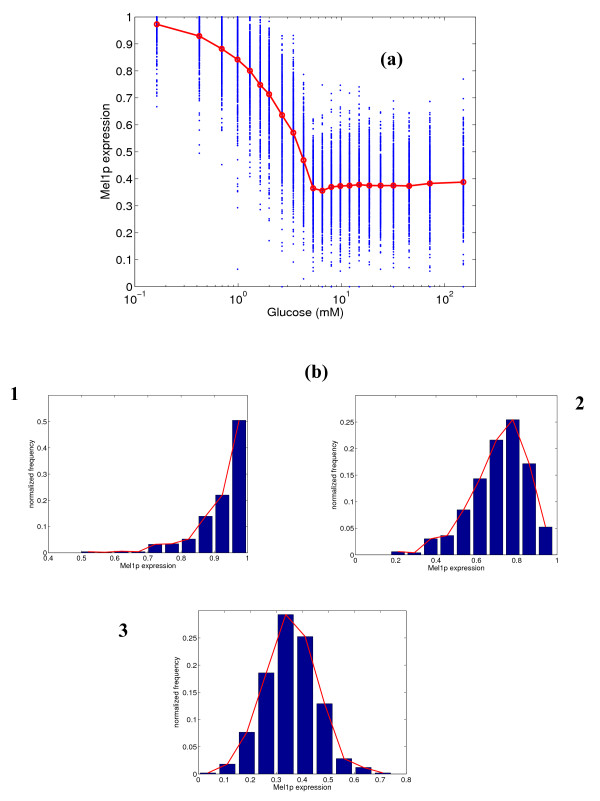
**(a) Simulated steady state protein expression for Mel1p at different glucose concentrations for the *in silico *mutant lacking the upstream activating sequence (UAS) for Gal4p, and (b) distribution of expression at 0.42, 2.0 and 6.55 mM glucose.** In figure (a), the blue dots represent the range of expression values over 500 simulations, and the red line represents the mean expression.

**Figure 8:**
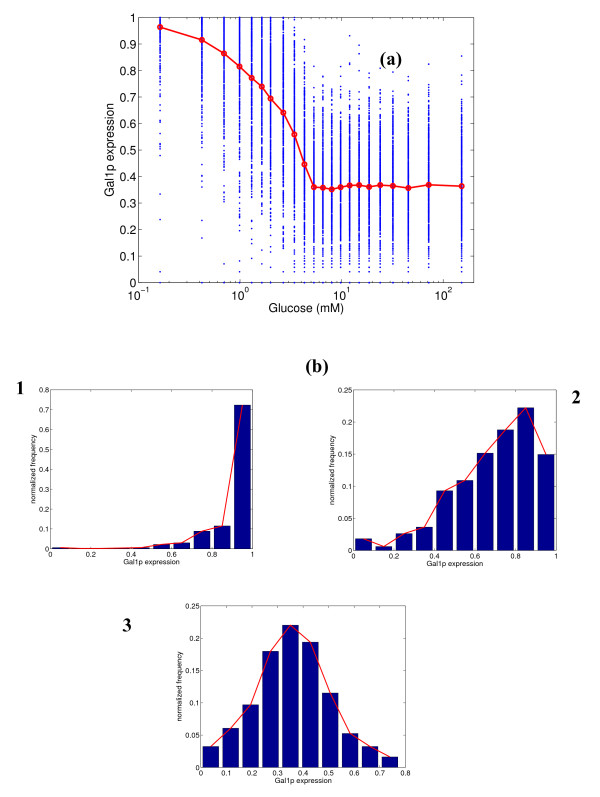
**(a) Simulated steady state protein expression for Gal1p at different glucose concentrations for the *in silico *mutant lacking the UAS for Gal4p, and (b) distribution of expression at 0.42, 2.0 and 6.55 mM glucose.** In figure (a), the blue dots represent the range of expression values over 500 simulations, and the red line represents the mean expression.

Since there are two mechanisms of repression of *GAL *genes, our next studies attempt to decipher the individual role of each of the repressors, Gal4p and Mig1p. Figure 7 shows the distribution of the *MEL1 *gene for an *in silico *mutant strain lacking the UAS for Gal4p. In such a strain, it can be observed from the figure that glucose cannot completely repress the expression of Mel1p. The degree of leakiness in the response is very high (mean = 40%). The histograms shown in panel 'b' demonstrate that there is high variability at all glucose concentrations. The variability at low glucose concentration (high expression) is comparable to or smaller than that in the original strain (see Figure 2), but the variability at other concentrations has increased. Figure 8 shows the expression of Gal1p for the same *in silico *mutant strain lacking Gal4p. The expression is again very leaky (mean = 35%) at high concentrations, and glucose cannot completely repress the expression of Gal1p, either. An important observation is that the switch-like behavior of Gal1p is lost, and the variability is higher at all concentrations, but especially so at higher glucose levels. This implies that Gal4p is essential for complete repression of *GAL *genes and to obtain crisper switching between the expression states.

We have also conducted simulations for the corresponding expressions of these genes in another *in silico *mutant strain lacking the URS for Mig1p. The expression is similar to that observed for the original *GAL80 *mutant strain, with marginally higher variability (Figure S1 in Additional file 1). The leakiness and variability are obviously lower than in the other *in silico *mutant strain lacking the UAS for Gal4p. In the case of the *in silico *mutant strain lacking the URS for Mig1p, η_H _is 2.9 for *GAL1 *expression and 1.7 for *MEL1*. This indicates that Mig1p imparts a part of the sensitivity seen in the original *GAL80 *mutant strain. The *in silico *mutant strains analyzed did not demonstrate any significant change in the half saturation constants as compared to the original *GAL80 *mutant strain. These results indicate that Gal4p is a more dominant regulator than Mig1p for these two genes.

**Figure 9:**
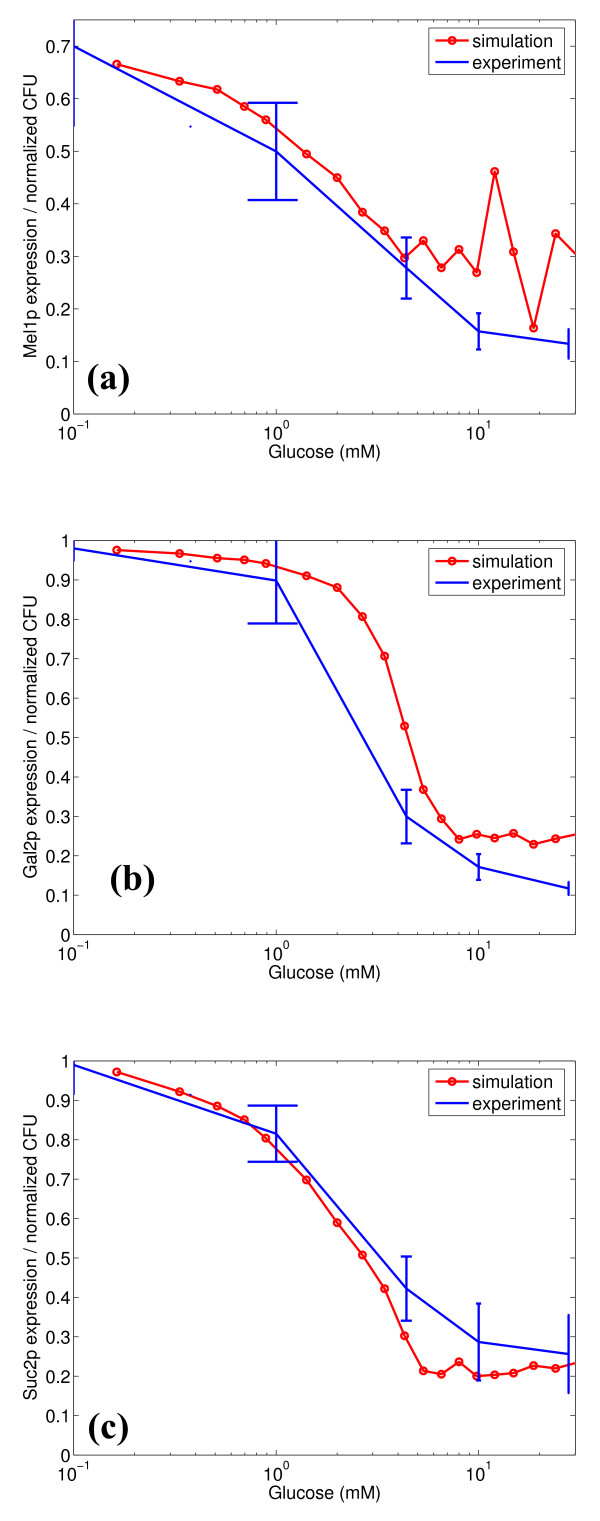
Comparison of variability in colony forming units (CFU) with variability at the protein expression level obtained through simulations for growth on (a) melibiose (Mel1p), (b) galactose (Gal2p), and (c) sucrose (Suc2p).

The above simulation results demonstrate that stochastic effects in the gene expression are significant in the *GAL *system for the *GAL80 *mutant strain of *S. cerevisiae*. A relevant question that can be raised at this point is whether this stochastic variability is transmitted to the phenotypic level of growth. To investigate this, experiments were conducted using a *GAL80 *mutant strain precultured on different glucose concentrations (see details in methodology). The precultured cells were streaked on plates containing melibiose (for *MEL1 *response), galactose (for *GAL2 *response) and sucrose (for *SUC2 *response). Figure 9 overlays the mean and variability (represented by the standard deviations) of the normalized number of colonies formed on the plates on the simulated distributions from Figures 2, 4 and 5. It must be noted that we have normalized the mean expression of the simulations based on the simulations yielding higher than 5% protein expression. This is to ensure a fair comparison with the experimental data. The normalization for the experimental CFU (based on the maximum CFU formed at steady state) only takes into account cells that have expressed and grown, and the normalization described above places the simulation results on the same basis. This is because we assume that the cells with lower than 5% expression do not grow. For all the three substrates, the simulation trends of the response were in close agreement with the colonies experimentally observed. For the melibiose plates, the variability in the colonies formed was high for cells precultured at low glucose concentrations, and reduced for cells precultured on high glucose concentrations. This is in agreement with the trends for the expression of *MEL1 *predicted by the simulations. For the galactose plates, the cells precultured at intermediate glucose concentrations (1 mM) have the largest variability, and the variability reduces significantly in both directions. For the sucrose plates, the variability in colonies formed increases slightly in the direction of increasing precultured glucose concentration.

**Figure 10:**
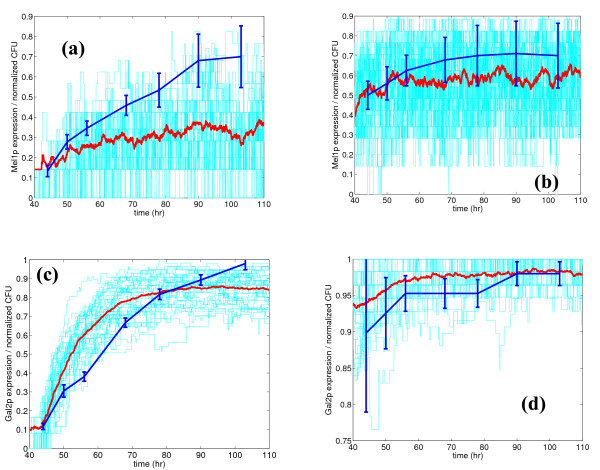
**Dynamic experimental data (44 – 100 hrs) for growth (in normalized CFU) on melibiose (Mel1p) and on galactose (Gal2p), along with simulations of Mel1p and Gal2p expression, at two glucose concentrations (1 mM and 27.8 mM).** Figures (a) and (b) show results for Mel1p at 27.8 mM and 1 mM, respectively. Figures (c) and (d) show the results for expression of Gal2p at 27.8 mM and 1 mM, respectively. Light colored lines show results of individual simulations, the solid line shows the mean of these simulations, and the dashed line with error bars shows the experimental data.

Figure 10 shows the dynamic progression of the colonies formed on the plates with melibiose and galactose. Since the plates do not contain any glucose, the variability in the final steady state colonies formed should indicate the low glucose concentration limit of the steady state gene expression predicted by the simulations. Figures 10a and 10b show the dynamics for the melibiose plates for preculturing at 27.8 mM and 1 mM glucose, respectively. Figures 10c and 10d show the dynamics for the galactose plates at 27.8 mM and 1 mM, respectively. The trends in expression predicted by simulation match with the experimental trends in normalized CFU. The mean expressions of the simulation also fall within the error bounds in the experimental data, except for cells grown on melibiose after preculturing at 27.8 mM glucose concentration. The dynamics are shown only from 44 hours onwards, since this is the time at which the first colonies became visible. Counting can obviously not be done prior to this time. Also, simulated profiles were shifted by 40 hours to account for this lag. Similar to the mean, the variability in the simulated expressions (light colored lines in Figure 10) also shows the same trends as the variability in experimental CFU. For *MEL1*, the variability increases with time for both preculturing states, which is similar to the trend observed (in steady state) in Figure 9(a) with decreasing glucose concentration. For *GAL2*, variability does change appreciably with time for preculturing at 27.8 mM glucose. However, the variability is high at lower times, and decreases significantly with time at 1 mM glucose. The high variability at lower times matches with the high variability observed at intermediate glucose concentrations (similar to 1 mM) in Figure 9(b), which was observed to be due to the steepness of the response curve at those concentrations.

## Discussion

*S. cerevisiae *is capable of growing on various carbon sources in its natural habitat. The organism prefers to grow on glucose in the presence of other carbon sources such as galactose, melibiose and sucrose. This requires the existence of a transcriptional mechanism to regulate the uptake of the other sugars. This transcriptional mechanism is known and well-studied [35] in yeast. Specific to the glucose regulation of the uptake of the three carbon sources (galactose, melibiose and sucrose), two mechanisms have been identified. For example, Mig1p, a repressor activated by glucose, and Gal4p, a transcriptional activator inhibited by Mig1p, are independent regulators in the regulation of *MEL1 *(for melibiose). In this case, the *MEL1 *gene has one binding site for both Mig1p and Gal4p. Also, for the regulation of *GAL1 *(for galactose), there are two binding sites for Gal4p and one for Mig1p. In the case of *SUC2 *(for sucrose), there are two binding sites for Mig1p, and the regulation is independent of Gal4p. Thus, the regulation of *SUC2 *is controlled by only one mechanism, but with two binding sites. These varied mechanisms and their hierarchy allow the organism to efficiently utilize and switch from one carbon source to the other [20,36-38].

Our current study provides insights into the stochastic effects of the various mechanisms described above on the expression of *GAL *and *SUC2 *genes. The analysis clearly demonstrates that the glucose repression on the uptake of other sugars is indeed noisy, resulting in high variability in the gene expression. Furthermore, the stochastic noise is directly dependent on the mechanism prevailing for a specific gene. The conservation of signal amplification and sensitivity observed in the steady state analysis was also confirmed in our stochastic simulations. For *MEL1*, the variability is high at low glucose concentrations, and lower at high glucose concentrations. For *GAL1*, the variability is lower than that for *MEL1 *at both extremes of glucose concentration. The only difference in the mechanism of glucose repression of *GAL1 *and *MEL1 *is the presence of an extra Gal4p binding site on *GAL1*. This additional binding site essentially helps in lowering the variability for *GAL1 *expression. This result is similar to one described in [24], where it was shown that basal transcription levels (and variability) for gal7p were reduced with extra binding sites. Thus, the presence of both the repression mechanisms for expression of Gal1p leads to a switch-like response to glucose, with the expression residing either in the completely expressed or repressed states. Gal2p, which only has binding sites for Gal4p, shows a steep response curve; however, there is considerable variability at intermediate glucose concentrations, and the response is not switch-like as for Gal1p. This implies that the repression mechanism involving Mig1p plays a role in establishing a switch-like response in protein expression. A possible reason for the absence of Mig1p in the regulation of Gal2p is that the galactose uptake must be metabolized in a graded manner. Gal1p, which is downstream of Gal2p in the metabolic pathway, catalyzes intracellular galactose and ATP to galactose-1-phosphate, thus determining the amount of intracellular substrate and the energy status. This makes Gal1p a crucial enzyme in the Leloir pathway of galactose uptake, as it determines whether the pathway is switched on or not. Also, the expression of Gal2p is leaky at high glucose concentrations, indicating that the system is ready for galactose uptake as soon as Gal1p expression switches on in response to the absence of glucose. For expression of Suc2p, the response is similar to that for Gal2p in terms of variability and leakiness. However, the steepness of the response curve is lower than that of Gal2p. *SUC2 *is regulated only by Mig1p with two binding sites, and the higher sensitivity associated with the Gal4p repression mechanism is not seen here. Since sucrose is a carbon source not linked to the galactose metabolic pathway, it may have evolved to be regulated only by Mig1p, so as to provide a graded response. Gal4p expression is leaky at high glucose concentrations, as it is a global transcriptional activator and needs to be available to switch on the system. There is variability at all glucose concentrations, which is the result of having only one binding site for Mig1p. Tight regulation of Gal4p is anyway not essential, since all that is required is a graded response to glucose.

The roles of the various mechanisms were also investigated by simulating the stochastic model on *in silico *mutant strains generated by eliminating individual mechanisms. The results clearly indicate that both the mechanisms are necessary for complete repression at high glucose concentrations. The mechanism involving Gal4p is a more dominant mechanism to regulate noise and stochastic effects than the mechanism involving Mig1p. However, at low glucose concentrations, the two binding sites present for Mig1p are sufficient to lower the noise in the expression. This is in contrast to the expression of *MEL1 *(which has only one binding site for Mig1p) at low glucose, which shows high variability. To conclude, the study indicates that multiple mechanisms tightly regulate the variability and expression at high glucose concentrations, while multiple binding sites for the regulators control the variability at lower glucose concentrations. The motif of multiple regulatory mechanisms having a role in reducing variability has been observed in simulations on the Gal3p and Gal80p mechanisms in a wild type strain of *S. cerevisiae *[24,25].

We also conducted growth experiments on agar plates to investigate the variability at the phenotypic level. Such experiments would indicate if the noise introduced at the transcriptional level is transmitted to the phenotype. Our experiments indicate a similar trend in the variability in growth as that of the simulated gene expression for all the three substrates. For example, high variability was observed in the *MEL1 *expression in the simulations at low glucose concentrations. This was also observed in the growth experiments on melibiose for cells precultured on low glucose concentrations. The dynamics of the growth experiments also confirmed that the cells demonstrate similar variability as observed in simulations, and tend towards the variability observed in the low glucose limit with time.

Specific mechanisms utilized by the cell to regulate expression of different genes responsible for the uptake of various carbon sugars by glucose demonstrate different levels of noise. The hierarchy in the variability introduced in the transcriptional mechanism sets up a corresponding hierarchy in the uptake of different sugars. In the case of glucose repression, the variability is highest for sucrose at high glucose concentrations, followed by galactose and melibiose. This results in sucrose being taken up before galactose and melibiose. However, at low glucose concentrations, the variability observed for growth on galactose was lower than that observed for melibiose, resulting in galactose being taken up before melibiose. Thus, the prevailing mechanisms result in a hierarchical uptake of sugars, in the order glucose, sucrose, galactose and melibiose.

Thus, the different mechanisms demonstrated different noise characteristics at the gene expression level, and this differentiation was carried through to the phenotypic level of growth. This may have important implications on the understanding of the effect of 'intrinsic' and 'extrinsic' noise [1,2] in the glucose repression in the regulation of *GAL/SUC2 *genes. The experiments showed the same trends as the simulations, but with slightly lower variability, possibly implying modulation of the noise through metabolism and cell division, leading to the phenotypic response.

## Conclusion

Transcriptional regulation involves protein-DNA interactions, and these involve reactants that are present in low concentrations, leading to the presence of stochasticity. This stochasticity may influence the phenotypic response of an organism. We have demonstrated that the stochasticity at the transcriptional level in glucose repression on the uptake of other substrates in yeast is transmitted to its growth. This implies that the intrinsic noise propagates through the metabolism and growth. The mechanisms involved in the transcriptional regulation and their variability set up a hierarchy in the phenotypic response. More experiments are needed in a single cell to measure the variability at the transcriptional, translational and metabolic levels. Further, studies on mutants obtained by disrupting specific mechanisms will provide more insights into the relationship between mechanisms and stochasticity. Studies on other transcriptional regulation systems and organisms are needed to generalize the relationship between noise and the phenotypic response. Finally, simulations incorporating models of metabolism [27] and dynamic experiments elucidating transitions between protein distributions [39] will provide a quantitative link between the genetic and the phenotypic levels.

## Methods

The schematic of the *GAL *network in the mutant strain of *S. cerevisiae *that we consider for the stochastic modeling is shown in Figure 1. Extracellular glucose is first transported into the cell, and then dephosphorylates Mig1p in the cytoplasm. The dephosphorylated Mig1p is transported into the nucleus, where it binds to various URS for the *GAL *and *SUC2 *genes. It should be noted that SUC2 has two URS for Mig1p, while *GAL1*, *GAL4 *and *MEL1 *have one each. The Gal4p synthesized interacts with the UAS of the *GAL *genes. In this case, *MEL1 *has one binding site for Gal4p, while the remaining seven *GAL *genes, including *GAL1 *and *GAL2*, have two binding sites. To reiterate, glucose represses the *GAL *and *SUC2 *genes by two mechanisms – by recruiting the repressor Mig1p into the nucleus, and by repressing the synthesis of the transcriptional activator Gal4p. These mechanisms were incorporated into our stochastic model to obtain insights on their relative importance.

### Simulation

We assume a Mig1p concentration in the nucleus as an input to our stochastic model. This nuclear Mig1p is related to the extracellular glucose (Glu) through a steady state Michaelis-Menten type relationship as given below:

(1) $$\frac{Mig1p}{Mig1p_{\max}}=\frac{Glu}{K_{s}+Glu}$$

where Mig1p_max _is the maximum concentration of Mig1p in the cell (assumed to be equivalent to approximately 100 molecules), and K_s _is the half-saturation constant. We then consider all the interactions described above (see Figure 1), and include them in our model (Additional file 1 lists all the species and reactions considered in our simulations). We consider the binding of the repressor, nuclear Mig1p, and Gal4p, to the respective binding sites as reversible stochastic reactions. The stochastic rate constants used to compute the propensities for each of the reactions (forward and backward) are estimated by the following procedure: First, the deterministic dissociation constants for the reversible reaction are obtained from the literature [28,29], the forward rate constants are estimated using information from the dynamic deterministic model of Ruhela et al. [29] where available, and the backward rate constants are set to satisfy the relation between rate constants and dissociation constants. For those reactions for which forward rate constants are not available, the values were set to match predicted expression to the mean steady state profiles obtained by Verma et al. [30]. We have included reactions that represent the transcription process; specifically, the binding of RNAP to the promoter site. Further, to quantify the translation, we assume that a fixed logarithm of fold change in mRNA would yield a net logarithm fold change in protein concentration.

(2) log_10_(Δ*p*) = *x*log_10_(Δ*mRNA*)

Δ*p *represents the fold change in protein expression, and Δ*mRNA *represents the fold change in mRNA expression, while *x *is the co-response coefficient [31] of protein expression and mRNA. It has been reported that the value of *x *is about 0.3 when all the mRNA is translated to protein in *S. cerevisiae *[32]. The value of *x *has been recalculated as 0.5 for *GAL *genes from the data of Ideker et al. [17]. In terms of fractional translation, the fractional protein expression can be related to the fractional transcription as follows,

(3) *f*_*p *_= *f*^*x*^

where *f*_*p *_and *f *are the fractional protein expressed by a gene and the fractional mRNA synthesized, respectively.

We have accounted for the change in cell volume and concentrations of components during cell growth and division by assuming a dilution effect on all the components through a simple first order degradation rate. The deterministic equation representing this is integrated along with the stochastic reactions using the methodology of Haseltine and Rawlings [33].

Simulations were also carried out for *in silico *mutant strains wherein the Mig1p binding sites were deleted from the *MEL1 *and *GAL1 *genes. Also, simulation studies were carried out with elimination of Gal4p binding sites for these two genes. These simulations help in determining the extent of repression through these two mechanisms.

All the simulations were performed using the direct method of the stochastic simulation algorithm (SSA) of Gillespie [5,7]. For the *GAL *system, the SSA provided results in reasonable time; thus, using approximate algorithms (e.g., tau-leaping) to speed up the simulation was not necessary. Since the system is stochastic, each run is a particular realization of the true dynamics of the system. Thus, the results over multiple (500) runs in an ensemble were averaged to obtain the mean values and distributions of the component populations. Past studies on steady state *GAL *gene response to glucose concentrations [22] have demonstrated that the sensitive range of glucose concentrations in which the *GAL *system is responsive is approximately 0.1 mM to 15 mM. This range was used to set the initial condition for the equivalent Mig1p nuclear concentrations using Equation (1).

### Experiments

#### Yeast Strains

*GAL80 *mutant strain of *S. cerevisae *Sc285 with genotype MATa ura3-52 leu2-3 2-112gal80 [34] was used in the study. It should be noted that Sc285 strain contains natural *MEL1 *in its genome, and can grow on melibiose as a carbon source.

#### Inoculum

The strains were stored in 20% (v/v) glycerol at -80°C in micro centrifuge tubes. The cells were precultured in YPD broth and streaked out onto YPD plate, from which a single colony was picked up to inoculate the shake flask.

#### Medium for the preculture

A cotton-stoppered, 500 ml Erlenmeyer flask containing 100 ml medium of following composition: 25 mg/L adenine, 5.0 g/L Yeast extract, 10.0 g/L peptone and 30.0 g/L glycerol was used. The pH was adjusted to 5.5 with 1 M HCl. The cells were grown in a shake flask at 240 rpm on a rotary shaker at 30°C for 12–16 h, until the cell concentration reached 1.0–1.5 OD at 600 nm. Subsequently, the bioreactor was inoculated with 10% cell mass of OD 1.0 at 600 nm.

#### Cultivation Conditions

Initially, *S. cerevisae *was grown in a batch bioreactor until biomass reached about 0.5–0.85 OD at 600 nm in a medium of composition 25 mg/L adenine, 5.0 g/L Yeast extract, 10.0 g/L peptone and 30.0 g/L glycerol. After this, the bioreactor was operated in a fed-batch mode by maintaining different average glucose concentrations (± 10%). The glucose concentration in the reactor was maintained by continuous feeding of standard glucose solution using calibrated peristaltic pumps (Watson Marlow 101U) through a feedback control mechanism. Different average glucose concentrations (with a set point for each) were maintained by altering the feed rate and the concentration of standard glucose solution. Steady state glucose concentration was thus maintained by feeding two standard glucose solutions (10 and 100 fold of required concentrations) by using peristaltic pumps. The glucose concentrations maintained in the fed-batch reactor were 0, 1, 4.4, 10 and 27.8 mM.

#### Plate Experiments

The inoculums from the fermentor, on reaching a fixed steady state glucose concentration, were streaked onto plates containing three different carbon sources, melibiose, galactose and sucrose at 20 g/L. The colonies were counted as colony forming units (CFU) beginning from 44 hours up to the time that the colonies reached a steady state number. It should be noted that the colonies were counted for different preculturing states depending on the glucose concentration used for their growth in the fed-batch reactor. Ten experiments with three sets in each of these experiments were carried out. Thus, the data is presented as a mean of thirty plates with their respective standard deviations, normalized with respect to the maximum number of colonies formed on the individual plates.

## Authors' contributions

VP and KVV conceived the modeling and experimental study, developed the models and experimental protocol and conducted all the analysis together.

## Supplementary Material

Additional File 1 **Supplementary information.** Lists (1) the initial conditions used in the simulations. (2) the reaction scheme and parameter values used in the simulations. (3) Figure S1, expression for Gal1p for *in silico *mutant lacking Mig1p. Click here for file
