# Supplementary material for: Stochastic analysis of the GAL genetic switch in Saccharomyces cerevisiae: Modeling and experiments reveal hierarchy in glucose repression
Source: BMC Syst Biol. 2008 Nov 17;2:97. doi: 10.1186/1752-0509-2-97 (PMC2614938; doi:10.1186/1752-0509-2-97)
Supplement: Additional File 1 — Supplementary information. Lists (1) the initial conditions used in the simulations. (2) the reaction scheme and parameter values used in the simulations. (3) Figure S1, expression for Gal1p for in silico mutant lacking Mig1p. [file 1752-0509-2-97-S1.doc]

**SUPPLEMENTARY INFORMATION**

**Kinetic model of the GAL genetic network in a mutant strain of *Saccharomyces cerevisiae* lacking GAL80**

| **Species** | **Description** | **Representative initial condition (molecules per cell)** |
| --- | --- | --- |
| GAL4 | Transcriptional activator Gal4p | 10 |
| (GAL4)_2_ | Gal4p dimer | 0 |
| D1 | *MEL1+GAL3* genes | 2 |
| D2 | All *GAL* genes with two binding sites (except for *GAL1*) for Gal4p | 6 |
| D1-(GAL4)_2_ | DNA-protein complex | 0 |
| D2-(GAL4)_2_ | DNA-protein complex | 0 |
| D2-(GAL4)_2_-(GAL4)_2_ | DNA-protein complex | 0 |
| D4 | *GAL4* gene | 1 |
| RNAP | RNA polymerase | 20 |
| D4-RNAP | DNA-RNAP complex | 0 |
| MIG1 | Transcriptional repressor Mig1p | 0-100 |
| MIG1-D4 | DNA-protein complex | 0 |
| DGAL1 | *GAL1* gene | 1 |
| DSUC2 | *SUC2* gene | 1 |
| D1-MIG1 | DNA-protein complex | 0 |
| DGAL1-MIG1 | DNA-protein complex | 0 |
| DGAL1-(GAL4)_2_ | DNA-protein complex | 0 |
| DGAL1-(GAL4)_2_-(GAL4)_2_ | DNA-protein complex | 0 |
| DSUC2-MIG1 | DNA-protein complex | 0 |
| DSUC2-MIG1-MIG1 | DNA-protein complex | 0 |
| D5 | All genes with binding sites for Mig1p (except D1, DGAL1, D4 and DSUC2) | 31 |
| D5-MIG1 | DNA-protein complex | 0 |

| **Index** | **Reaction** | **Kinetic expression** | **Description** | **Source for kinetic parameters** |
| --- | --- | --- | --- | --- |
| 1 | 2 GAL4 → (GAL4)_2_ | 6.5585e-8*[GAL4]*([GAL4]-1)/2 | Dimerization | Estimated from [28], [29] |
| 2 | (GAL4)_2_ → 2 GAL4 | 2.778e-4 *[(GAL4)_2_] | Dissociation | Estimated from [28], [29] |
| 3 | (GAL4)_2_ + D1 → D1-(GAL4)_2_ | 1.3170e-7* [(GAL4)_2_]*[D1] | Association | Estimated from [28], [29] |
| 4 | D1-(GAL4)_2_ → (GAL4)_2_ + D1 | 5.556e-5*[D1-(GAL4)_2_] | Dissociation | Estimated from [28], [29] |
| 5 | (GAL4)_2_ + D2 → D2-(GAL4)_2_ | 1.3170e-7* [(GAL4)_2_]*[D2] | Association | Estimated from [28], [29] |
| 6 | D2-(GAL4)_2_ → (GAL4)_2_ + D2 | 5.556e-5*[D2-(GAL4)_2_] | Dissociation | Estimated from [28], [29] |
| 7 | (GAL4)_2_ + D2-(GAL4)_2_ → D2-(GAL4)_2_-(GAL4)_2_ | 3.954e-4 [(GAL4)_2_]* [D1-(GAL4)_2_] | Association | Estimated from [28], [29] |
| 8 | D2-(GAL4)_2_-(GAL4)_2_ → (GAL4)_2_ + D2-(GAL4)_2_ | 5.556e-5*[D2-(GAL4)_2_-(GAL4)_2_] | Dissociation | Estimated from [28], [29] |
| 9 | D4 + RNAP → D4-RNAP | 1.1112e-3*[D4]*[RNAP] | Association | Estimated from [28], [30] |
| 10 | D4-RNAP → GAL4 + RNAP + D4 | 1.3890e-2*[D4-RNAP] | Gal4p synthesis | Estimated from [28], [30] |
| 11 | MIG1 + D4 → MIG1-D4 | 6.5585e-5*[D4]*[MIG1] | Association | Estimated from [28], [30] |
| 12 | MIG1-D4 → MIG1 + D4 | 5.556e-5*[MIG1-D4] | Dissociation | Estimated from [28], [30] |
| 13 | D1 + MIG1 → D1-MIG1 | 6.5585e-5*[D1]*[MIG1] | Association | Estimated from [28], [30] |
| 14 | D1-MIG1 → D1 + MIG1 | 5.556e-5*[D1-MIG1] | Dissociation | Estimated from [28], [30] |
| 15 | DGAL1 + MIG1 → DGAL1-MIG1 | 6.5585e-5*[DGAL1]* [MIG1] | Association | Estimated from [28], [29] |
| 16 | DGAL1-MIG1 → DGAL1 + MIG1 | 5.556e-5*[DGAL1-MIG1] | Dissociation | Estimated from [28], [29] |
| 17 | (GAL4)_2_ + DGAL1 → DGAL1-(GAL4)_2_ | 1.3117e-5*[DGAL1]* [(GAL4)_2_] | Association | Estimated from [28], [29] |
| 18 | DGAL1-(GAL4)_2_ → (GAL4)_2_ + DGAL1 | 5.556e-5*[DGAL1-(GAL4)_2_] | Dissociation | Estimated from [28], [29] |
| 19 | (GAL4)_2_ + DGAL1-(GAL4)_2_ → DGAL1-(GAL4)_2_-(GAL4)_2_ | 3.954e-4*[DGAL1-(GAL4)_2_]* [(GAL4)_2_] | Association | Estimated from [28], [29] |
| 20 | DGAL1-(GAL4)_2_-(GAL4)_2_ → (GAL4)_2_ + DGAL1-(GAL4)_2_ | 5.556e-5*[DGAL1-(GAL4)_2_- (GAL4)_2_] | Dissociation | Estimated from [28], [29] |
| 21 | DSUC2 + MIG1 → DSUC2-MIG1 | 6.5585e-5*[MIG1]* [DSUC2] | Association | Estimated from [28], [29] |
| 22 | DSUC2-MIG1 → DSUC2 + MIG1 | 5.556e-5*[DSUC2-MIG1] | Dissociation | Estimated from [28], [29] |
| 23 | DSUC2-MIG1 + MIG1 → DSUC2-MIG1-MIG1 | 6.5585e-5*[MIG1]* [DSUC2-MIG1] | Association | Estimated from [28], [29] |
| 24 | DSUC2-MIG1-MIG1 → DSUC2-MIG1 + MIG1 | 5.556e-5*[DSUC2-MIG1-MIG1] | Dissociation | Estimated from [28], [29] |
| 25 | D5 + MIG1 → D5-MIG1 | 6.5585e-5*[MIG1]*[D5] | Association | Estimated from [28], [29] |
| 26 | D5-MIG1 → D5 + MIG1 | 5.556e-5*[D5-MIG1] | Dissociation | Estimated from [28], [29] |
| 27 | GAL4 → | 3.3336e-5*[GAL4] | Degradation |  |

Time units for rates are seconds.

Supplementary Figure S1: (a) Simulated steady state protein expression for Gal1p at different glucose concentrations for the *in silico* mutant strain lacking the URS for Mig1p, and (b) distribution of expression at 0.33, 3.43 and 6.55 mM glucose.

**(b)**

| **1** | **2** |
| --- | --- |
| **3** | |
